# Imaging the PM/AICD patient; is it beneficial to the final diagnosis?

**DOI:** 10.1186/1532-429X-16-S1-T7

**Published:** 2014-01-16

**Authors:** June A Yamrozik, Mark Doyle, Ronald B Williams, Geetha Rayarao, Diane V Thompson, Moneal Shah, Robert W Biederman

**Affiliations:** 1 Cardiac MRI, Allegheny General Hospital, Pittsburgh, Pennsylvania, USA

## Background

Pacemaker/AICD imaging is currently being performed on patients in the MRI environment. A vigilant team consisting of the (Cardiologist, EP staff, and technologists) with close patient monitoring and supervision is making such an unmentionable procedure a success. However, is the diagnosis from imaging these patients adding valuable irrefutable information to merit such a risk?

## Methods

A total of 48 patients were imaged on a GE CV/i Excite Version 12, 1.5 T system (GE, Milwaukee, WI). 8 patients had an AICD, 7 had an AICD/Pacemaker, 2 had a single pacemaker lead, 2 REVO pacemakers and 29 patients had a traditional dual-chamber pacemaker. A specific criteria was followed for all the patients undergoing this procedure to scrutinize if the final diagnosis provided additional information in patient care. A checklist of three questions were gathered and answered after the final interpretation of the MRI. Does the diagnosis change? Does the MRI provide additional information to the existing diagnosis? Does patient management change? If 'yes' was answered any of the above questions it was considered that the MRI scan was of value to patient diagnosis.

## Results

Regarding the population, of the 48 patients imaged, 36 (75%) were neurology cases and 12(25%) were cardiac/vascular cases. After reviewing the results from the 36 neurology cases and comparing the results from prior studies (CT, angio, EEG and/or myelogram) 16 (45%) out of the 36 showed that the MRI not only provided additional information but changed the original diagnosis and in turn their course of medical treatment. 8 patients (22%) provided additional information to the diagnosis. Thus a total of 24 patients (67%) showed that the MRI scan was of value to the final diagnosis. In 12(33%) out of the 36 patients imaged MRI did not provide further information but confirmed the original diagnosis. The 12 cardiac cases were also compared to prior studies (heart cath, TEE, TTE and stress) and in 4 patients (33%) the MRI provided additional information to change the original diagnosis and also patient management. The remaining 8 (67%) showed that extra information was gathered by having the Cardiac MRI procedure. In essence, 100% of the cardiac population benefited by having an CMR done.

## Conclusions

The use of PM/AICD imaging in MRI remains controversial but as the lead/generator technology has improved, increased confidence in its use is found. Herein, we show that MRI procedures on carefully selected patients with pacemakers/AICD's are beneficial and substantially add valuable irrefutable information to patient diagnosis and management We propose that not only are Pacemakers/AICD's no longer forbidden in the MRI environment but they can be markedly efficient with life-altering and life-saving consequences.

## Funding

None.

